# Single-shot and single-sensor high/super-resolution microwave imaging based on metasurface

**DOI:** 10.1038/srep26959

**Published:** 2016-06-01

**Authors:** Libo Wang, Lianlin Li, Yunbo Li, Hao Chi Zhang, Tie Jun Cui

**Affiliations:** 1 School of Electronics Engineering and Computer Science, Peking University, Beijing 100871, China; 2 State Key Laboratory of Millimeter Waves, Southeast University, Nanjing 210096, China

## Abstract

Real-time high-resolution (including super-resolution) imaging with low-cost hardware is a long sought-after goal in various imaging applications. Here, we propose broadband single-shot and single-sensor high-/super-resolution imaging by using a spatio-temporal dispersive metasurface and an imaging reconstruction algorithm. The metasurface with spatio-temporal dispersive property ensures the feasibility of the single-shot and single-sensor imager for super- and high-resolution imaging, since it can convert efficiently the detailed spatial information of the probed object into one-dimensional time- or frequency-dependent signal acquired by a single sensor fixed in the far-field region. The imaging quality can be improved by applying a feature-enhanced reconstruction algorithm in post-processing, and the desired imaging resolution is related to the distance between the object and metasurface. When the object is placed in the vicinity of the metasurface, the super-resolution imaging can be realized. The proposed imaging methodology provides a unique means to perform *real-time data acquisition*, high-/super-resolution images without employing expensive hardware (e.g. mechanical scanner, antenna array, etc.). We expect that this methodology could make potential breakthroughs in the areas of microwave, terahertz, optical, and even ultrasound imaging.

High-resolution or super-resolution imaging is a long sought-after goal in various imaging applications, where both real-time data acquisition and low-cost hardware are desired. Three types of active imaging systems have been developed for data acquisition in the past decades, namely, the real-aperture (RA) system^1^, the synthetic-aperture (SA) system^2^, and the random-pattern-based computational imager^3^,^4^,^5^,^6^. The RA system composed of a large amount of antenna elements in a large aperture is more flexible in measurement modes but suffers from the big size, heavy weight, high power, and high expense of hardware. The SA system, by contrast, belongs to the category of single-sensor systems. The completion of imaging relies on the mechanical movement of a single sensor to form virtually a large scanning aperture and subsequent data post-processing.

Inspired by the compressive sensing (CS) theory proposed about one decade ago, the single-pixel imaging systems have been developed to reduce the number of measurements while keeping acceptable imaging quality at optical^3^,^4^, terahertz^5^,^6^,^7^ and millimeter-wave^8^ frequencies. The key idea behind these imaging systems is to project the object’s information into the single-pixel detector through a set of spatial light modulators (SLMs) with quasi-random patterns at a single frequency. Apparently, the CS-based imaging apparatus falls into the category of multi-shot and single-sensor systems. Usually, the number of required SLMs is in the order of the sparsity degree of the probed object, and a sparsity-driven convex optimization problem must be solved to retrieve the information of the probed object. The series of random SLMs are manipulated in a sequential manner, suffering from low-efficiency data acquisition.

Recently, several excellent contributions on single-sensor imaging have been made by using single-resonant metamaterials^9^ or multi-resonant metamaterials^10^,^11^, which are typically spatio-temporal dispersive, combined with a sparsity-regularized reconstruction algorithm to treating the sparse microwave scene. Since such dispersive metamaterial can produce random radiation patterns over a broad frequency band excited by a broadband source, this system works as a single-shot and single-sensor imager^9^,^10^,^11^. However, the sparsity-regularized computational reconstruction algorithm involved only treats the imaging of well-separated sparse objects in the sense of the Rayleigh criterion, hence it is a low resolution imaging system^10^. In a later contribution by the same group, the imaging resolution problem has been addressed^12^, in which four-time shots of electromagnetic waves are required to collect enough measurement data. Therefore, it is a multi-shot and single-sensor system. More recently, a similar concept of passive single-sensor imager composed of a fan-like metamaterial waveguide was presented for detecting well-separated sparse acoustic sources^13^. This design also requires solving a sparsity-regularized convex optimization problem, making it a passive single-sensor system for low-resolution imaging.

In this work, we propose the first broadband single-shot and single-sensor microwave imager for the super-resolution imaging with the aid of a spatio-temporal dispersive metasurface, capable of coupling the evanescent modes into the propagating modes. During the past decade, metamaterials and metasurfaces have shown great promises to control electromagnetic waves in flexible ways, as evidenced in the demonstration of a number of interesting devices and applications, such as ultrathin flat lenses^14^, analogy signal processing^15^, switchable plasmon coupler^16^, high-resolution hologram^17^, and various other functionalities^18^,^19^,^20^,^21^. Recently, we have demonstrated that a well-designed metasurface can make conversions among different wave components, either propagating or evanescent waves, resulting from the localized surface plasmon resonances^22^,^23^. In this sense, it is expected that the spatial information of the probed object can be sufficiently embodied into the data acquired by a single sensor fixed in the far-field region.

We demonstrate to get the acceptable image quality through the procedure of simple data processing such as the standard minimum least square reconstruction algorithm, instead of using the sparsity-promoted iterative reconstruction algorithms^3^,^4^,^5^,^6^,^7^,^8^,^9^,^10^,^11^. The efficient conversion of evanescent waves to propagating waves can be achieved, which enables the super-resolution imaging by even using one single far-field sensor along with a single-shot excitation. In fact, the desired imaging resolution can be obtained by adjusting the distance between the probed object and the metasurface. When the object is placed in the vicinity of the metasurface, the super resolution is achieved. We show that the broadband single-shot and single-sensor imager brings us an expedient far-field super-resolution imaging scheme, which is capable of producing an image with resolution up to 0.2*λ*. Moreover, we experimentally demonstrate that the single-shot and single-sensor microwave imaging system has the unique ability to perform real-time data acquisition, and is capable of generating high-resolution images without mechanical scanning or antenna arrays.

## Results

### Theory and analysis

**Figure 1:**
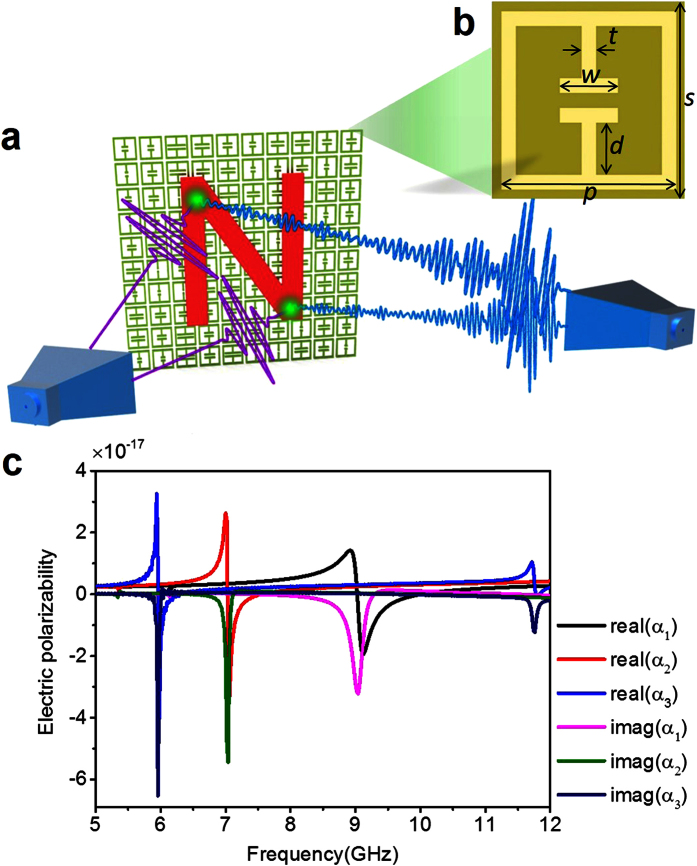
(**a**) The single-shot and single-sensor far-field imaging system based on a metasurface, which is composed of a cluster of strongly coupled subwavelength resonators. To efficiently convert the evanescent waves of the object under detection to the far field, the object should be in the vicinity of the metasurface. (**b**) The geometry of unit cell used to construct the metasurface, where *s* = 6.67 mm, *p* = 6 mm, *t* = 0.5 mm, *d* = 1.75 mm, and *w* varies from 0.6 mm to 4.8 mm to control the ELC resonant frequency. (**c**) The electric polarizabilities of unit cells retrieved from the method of dipole approximation when *w* = 1 mm, 2.6 mm, and 4.0 mm, respectively.

The configuration of the proposed single-shot and single-sensor imaging system is illustrated in Fig. 1(a). In this proposed system, a single shot of broadband electromagnetic wave (5–12 GHz) is illuminated on object under consideration, and a single far-field sensor fixed at ***r***_*d*_ is used to collect the wavefield scattered from object. A specially-designed metasurface is situated behind probed object, which is composed of a dielectric substrate board patterned with ELC resonators^24^ on both sides (*see Methods*). The substrate has a thickness of 0.5 mm and a dielectric constant of 4.3. The geometry of the ELC resonator is shown in Fig. 1(b). The resonant frequency is tuned by the parameter *w*. The dominant components (i.e., *y*-components) of the electric polarizability tensor for three typical values of *w* (1 mm, 2.6 mm, and 4.0 mm) are plotted in Fig. 1(c). The retrieval of these parameters is done in the commercial software, CST Microwave Studio 2014, following a general procedure^24^.

**Figure 2:**
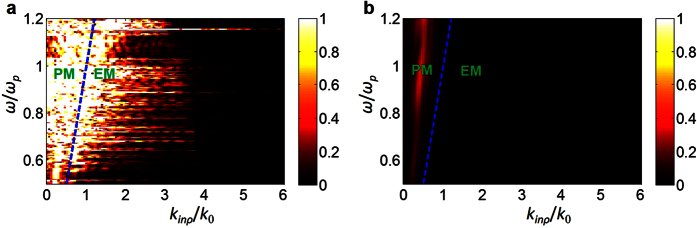
The amplitudes of electric fields recorded by a sensor in the far-field region, in which the horizontal axis represents *k*_*inρ*_ normalized to *k*_0_ (the free-space wavenumber at 10 GHz), while the vertical axis represents the frequency normalized to *ω*_*p*_ (the angular frequency at 10 GHz). (**a**)The metasurface is placed between the target and the sensor. (**b**)The metasurface is replaced by the FR4-material lens. The region on the left of the blue dashed line corresponds to the propagating modes (**PM**), while the region on the right of the blue dashed line corresponds to the evanescent modes (**EM**). These figures demonstrate that as opposed to FR4-material lens, the metasurface is capable of converting the evanescent waves into propagating waves.

The metasurface is of spatio-temporal dispersive, from which the three-dimensional spatial information of the probed object characterized by the reflectivity *O*(*x*, *y*, *z*) can be registered in the one-dimensional response *s*(*t*) acquired by one single sensor fixed at ***r***_*d*_. To provide more physical insights, we conduct a set of numerical simulations when the metasurface is illuminated by the transverse-electric (TE) polarized plane wave: ***E***_*in*_ = ***E***_0_ exp(*i**k***_*in*_ ⋅ ***r***), in which ***k***_*in*_ = (*k*_*inx*_, *k*_*iny*_, *k*_*inz*_), 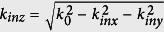, and *k*_0_ is the free-space wavenumber. The case of 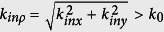 corresponds to the evanescent-wave illumination, while the case of *k*_*inρ*_ < *k*_0_ to the propagating-wave incidence. We perform the so-called coupled-dipole method^25^,^26^, a full-wave solver to Maxwell’s equations, based on the extracted electric polarizability tensors of the metasurface elements. The calculated amplitude of the scattered electric field at ***r***_*d*_ as a function of operational frequency and transverse wavenumber is plotted in Fig. 2(a), where the metasurface is supposed to consist of 30 × 30 ELC cells. For comparison, the corresponding result for the FR4-material lens in place of metasurface is reported in Fig. 2(b) as well. Here, the horizontal axis denotes the transverse wavenumber normalized by *k*_0_ (the free-space wavenumber at 10 GHz), while the vertical axis denotes the operational frequency normalized by *ω*_*p*_ (the angular frequency at 10 GHz). This map clearly demonstrates that different wave components can be transferred into the temporal domain after passing through the metasurface with substantially high efficiency; however, the FR4-material lens fails to accomplish this task. In this set of simulations, the metasurface is located at *z* = 6 mm (slightly off the plane of *z* = 0), in order to account for the decay effect of the evanescent waves. For the distance used in Fig. 2(a), the imaging resolution is expected to be around 0.2λ. Nevertheless, this distance can be tuned accordingly if different requirement on imaging resolution is adopted.

### Experimental results for high-resolution imaging

**Figure 3:**
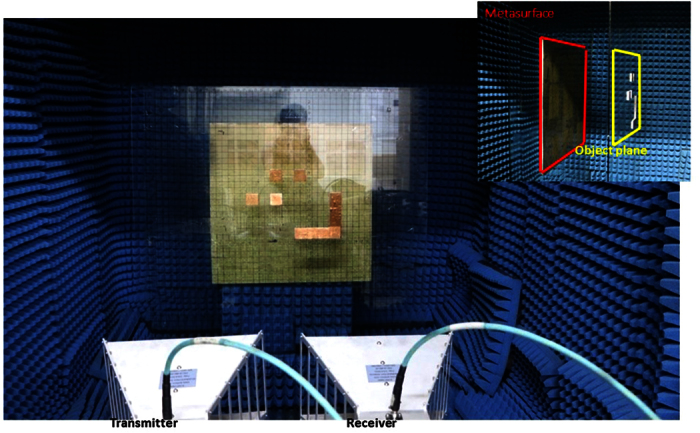
Experimental setup of the broadband single-shot and single-senor system for high-resolution and super-resolution imaging based on the proposed metasurface.

We provide selected experimental examples to validate the proposed single-shot and single-sensor system for high-resolution imaging (see Fig. 3). For this purpose, we fabricate a metasurface consisting of 30 × 30 ELC elements with random individual resonant frequencies. The metasurface is placed on the plane of *z* = 0. The parameters of the ELC elements are set as follows: *s* = 6.67 mm, *p* = 6 mm, *t* = 0.5 mm, *d* = 1.75 mm, and *w* is selected randomly from 12 values being 0.6 mm, 1 mm, 1.4 mm, 1.8 mm, 2.2 mm, 2.6 mm, 2.8 mm, 3.2 mm, 3.6 mm, 4.0 mm, 4.4 mm, and 4.8 mm.We use a horn antenna to launch the broadband single-shot excitation with the operating frequency ranging from 5 to 12 GHz with step of 4.375 MHz. Another horn antenna recording the electric-field responses scattered from the object at **r**_*d*_ = (0.3 m, 0.2 m, 1 m) is placed next to the transmitter. The experiments are carried out in a microwave anechoic chamber (*see Methods*). We present a point-to-point scanning technique to calibrate the knowledge of the measurement operator ***A***, which relates linearly the one-dimensional frequency-dependent response *s* measured by the single sensor to the investigated reflectivity *O* in the object plane.

**Figure 4:**
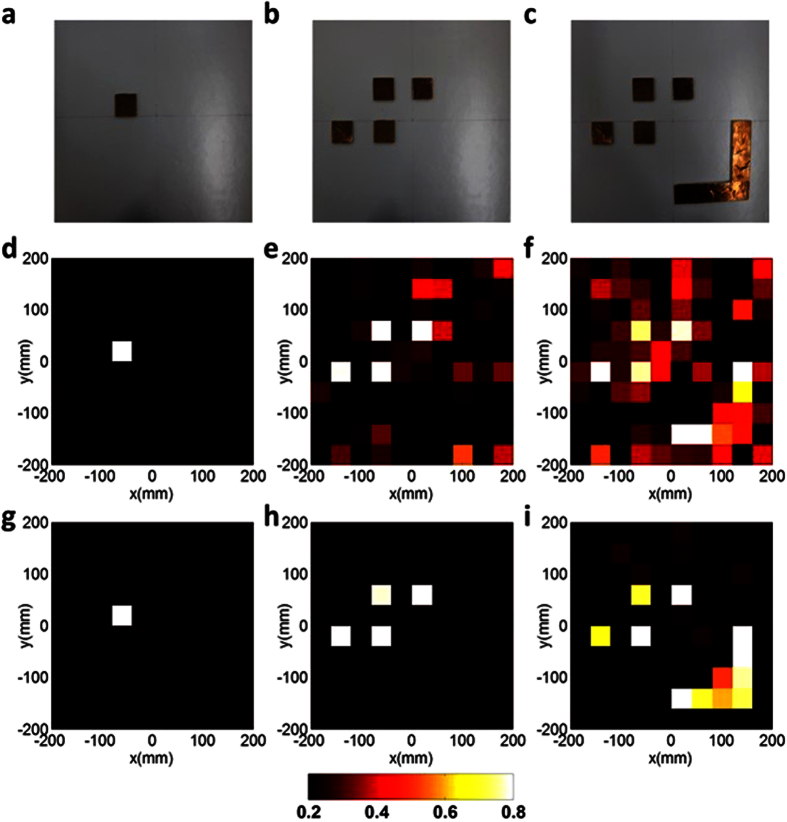
High-resolution imaging results of three metallic objects, in which a horn antenna is used to emit a short pulse, and the other horn antenna is used for receiving the time-dependent scattered fields at a fixed location. (**a–c**) Three metallic objects with increasing complexity, in which the distance between the objects and metasurface is set to be 0.3 m. (**d–f**) The imaging results using the traditional minimum least-square algorithm. (**g–i**) The imaging results using the iteratively reweighting algorithm. The imaging results demonstrate acceptable reconstructions of original objects, in which the use of advanced algorithm can further improve the imaging quality.

We demonstrate three typical scenes of metallic objects, shown in the top row of Fig. 4, which are placed in the object plane on z = 0.3 m, to investigate the performance of the proposed imaging method. Note that the complexity of the probed objects is successively increased from the left to the right. The corresponding reconstruction results of such objects normalized by their own maximums are provided in the middle row of Fig. 4, in which the standard minimum least square reconstruction algorithm is implemented to process the acquired one-dimensional temporal data. For computational reconstructions, the investigation domain has been divided into 10 × 10 = 100 uniform pixels, each of which has the size of 40mm × 40mm. From the middle row of Fig. 4, the reconstructed images with acceptable quality have been observed in the sense that the location and shape of objects under investigation can be clearly identified, especially for the object with relatively lower complexity, although some artifacts appear for the high-complexity object (e.g., Fig. 4(c)). It should be emphasized that the Born or single-scattering assumption has been explicitly made in our linearized imaging model, which neglects the multiple interactions among the metasurface and objects, and thus the results are relatively serious deterioration in terms of imaging quality for the high-complexity object due to the coupling effect, as illustrated in the rightmost column of Fig. 4.

In the past decade, the reconstruction algorithms have made significant progress owing to the revolutionary advances in signal processing, in which the task- or instance-driven prior on the underlying object has been incorporated into the imaging process to improve the imaging quality substantially^27^,^28^,^29^,^30^,^31^,^32^. Such advanced reconstruction algorithms refresh people’s minds revolutionarily on many things that were thought to be impossible, such as the Ankylography^33^, listening the shape of a room using a single microphone^34^. Here, considering the fact that the probed object is spatially sparse, we perform the *l*_1_-norm regularized reconstruction technique, more specially, the iteratively reweighting algorithm^23^,^30^ (*see Methods*). The reconstructed results of the three examples using the advanced algorithm are presented in the bottom line of Fig. 4. As expected, the imaging quality by incorporating the sparsity *prior* into the imaging procedure has been refined remarkably in comparison to those without using the sparse prior. Nonetheless, whether the use of sparsity or not in the reconstruction procedure, the imaging results of the probed objects are acceptable, showing the effectiveness of the proposed method. Hence the broadband single-shot and single-sensor system is sufficient to produce satisfactory imaging results against experimental data.

### Simulation results for subwavelength imaging

**Figure 5:**
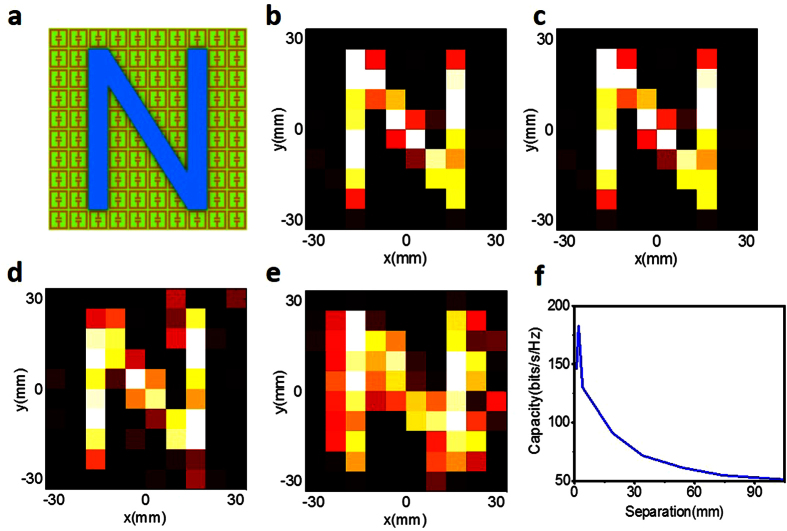
Far-field subwavelength imaging results, in which we emit the single broadband illumination using a horn antenna and receive the time-dependent scattered fields in the far-field region by the other single horn antenna. (**a**) An N-shaped dielectric object located near the metasurface. (**b**) The imaging results using the traditional minimum-least square algorithm. Here, an imaging resolution around λ/5 has been achieved. In (**c–e**), the distance between the object and metasurface is 4 mm, 10 mm, and 20 mm, respectively. (**f**) The dependence of sensing capacity on the distance between target and metasurface is plotted. The imaging quality is monotonously decreased as the distance of the object away from metasurface grows up.

Far-field imaging beyond the diffraction limit is desirable in various fields. In this pursued topic, three research streams have gained intensive attentions, including the super-oscillation^35^,^36^,^37^,^38^, the resonant structure^25^,^26^,^39^,^40^,^41^, and the structure-enhanced signal post-processing^30^ approaches. The essential requirement on such techniques is the sensor array or mechanical movement, which typically requires expensive hardware and time-consuming data acquisition. As demonstrated in Fig. 2, the metasurface is capable of converting the evanescent waves emerged from the object in the small vicinity of metasurface into the propagating waves, and thus allows the reconstruction of subwavelength-scale information of the objects by using a single sensor fixed in the far field. To verify this feasibility, a set of simulations are performed with the commercial software, CST Microwave Studio 2014. We use the same metasurface as studied above with 10 × 10 ELC elements possessing random resonant frequencies, and place a specimen in the vicinity of metasurface, namely, with a distance of 4 mm away from metasurface. The specimen under investigation is an capital character “N” made of dielectric bars with the refractive index of 4.3 and rectangular cross section of 0.5 mm × 6.67 mm. We voluntarily opt for a low-refractive-index contrast, standing for the soft-matter object. Again, we have calibrated the knowledge of the linear measurement operator in the point-to-point scanning manner, so that the one-dimensional temporal response acquired by the single sensor fixed at ***r***_*d*_ is linearly related to the imaged pixels in the object plane. In this study, the white noise with signal-to-noise ratio (SNR) 30 dB is added to CST simulation data, and the size of imaging pixel is taken as the ELC size (i.e., 0.2λ × 0.2λ). We plot the imaging results in Fig. 5(b), reconstructed from the traditional minimum least-square reconstruction algorithm, respectively, which show that the two objects separated by a distance of 6.67 mm (about 0.2λ) can be clearly resolved from far-field single-sensor measurements. Despite the simplicity of this example, we demonstrate that the subwavelength-scale information of an object can be registered in the far-field region by using the broadband illumination and a spatio-temporal metasurface. Moreover, we verify that an object which is not aligned with the metamaterial lattice can also be imaged. We underline here that such super-resolution imaging can be realized in real time, as it only requires a single-shot illumination and single-sensor data acquisition from the far field, removing the harsh requirements of sensor array, mechanical movement, and near-field measurements.

## Discussion

The concept of broadband single-shot and single-sensor imager relies on the key point that the metasurface could encode the spatial structure of the object into the temporal signal acquired by a single sensor fixed in the far-field region. Consequently, the imaging resolution depends largely on the distance between the metasurface and investigated object. To demonstrate this issue clearly, a set of numerical simulations are performed using CST Microwave Studio 2014, where the imaged object is the same as that in Fig. 5(a). We investigate how the imaging quality (or resolution) is affected by varying the distance between the metasurface and the object. Fig. 5(c–e) show three reconstructed images of the object for three representative distances between the metasurface and object, in which the simulation parameters are the same as those adopted in Fig. 5(b) and the standard minimum least-square reconstruction algorithm is applied for data processing. A more comprehensive examination is provided in Supplementary Information (*see Video*). From Fig. 5(c–e), we observe that the imaging quality will become worse as the distance between the object and metasurface gets larger, since for the relatively bigger distance the evanescent wave carrying the information of the finer structures of the probed objet will vanish before arriving at the metasurface, and thus cannot be captured by the single-sensor in the far field. The subwavelength imagining considered in our work is for weak object such that Born approximation can be made. Therefore, when the object is very close to metasurface, the imaging quality will be distorted due to coupling effect, as illustrated in video. To quantify the amount of information that is gained when the target is located closer to the metasurface, the dependence of sensing capacity^42^ on the distance between target and metasurface is plotted in Fig. 5(f). From Fig. 5(f), one can assess the gain resulted from coupling the evanescent into the radiative modes by the use of the metasurface.

In conclusion, we have presented a broadband single-shot and single-sensor system for high-resolution and subwavelength imaging, in combination with the metasurface that can convert the spatial information of the probed object into the measured temporal data by a single sensor. We demonstrated that such an imaging system has the unique ability to achieve high-resolution and super-resolution images in real-time manner without using sensor array as well as mechanical movement and near-field scanning of sensor. We expect that the proposed methodology could make potential breakthroughs in the imaging technologies in microwave, terahertz, optical, and ultrasound regimes.

## Methods

### Experiments

We fabricate the designed metasurface using a commercial FR4 substrate, of which both sides are patterned with ELC resonators. The FR4 substrate has a thickness of 0.5 mm, a dielectric constant of 4.3, and a loss tangent of 0.002. The parameters of the ELC resonant elements are set as follows: *s* = 6.67 mm, *p* = 6 mm, *t* = 0.5 mm, *d* = 1.75 mm, and the value of *w* is selected randomly from 12 of values being 0.6 mm, 1 mm, 1.4 mm, 1.8 mm, 2.2 mm, 2.6 mm, 2.8 mm, 3.2 mm, 3.6 mm, 4.0 mm, 4.4 mm, and 4.8 mm. In our experiments, we use a pair of broadband horn antennas, one plays the role of broadband single shot with working frequencies ranging from 5 to 12 GHz to launch broadband illumination, and the other serves as the single sensor for receiving the phase and amplitude responses of the electric field (see Fig. 3). A vector network analyzer (VNA, Agilent E5071C) was used for the experimental far-field imaging, and the whole experiment is carried out in a microwave anechoic chamber with the size of 2 m × 2 m × 2 m. The horn antennas are connected to the two ports of VNA through 4 m-long and 50-Ω coaxial cables. The measured S_21_ parameters (i.e., transmission coefficients) are utilized as the data input for the imaging reconstruction.

The calibration of the mapping matrix ***A*** is accomplished by the point-to-point raster scanning method, i.e., sequentially moving a small object (a small Chinese coin) followed by recording the corresponding S_21_ responses. This process is repeated until all imaging pixels are completely scanned. To suppress the measurement noise level, the average number and filter bandwidth are set to be 7 and 1 kHz in VNA. In addition, we have also performed the experiments on subwavelength imaging, where the probed object is similar to the first two specimens used in the high-resolution situation, but with 0.1 times scale, and the probed object is composed of FR4 materials. Results visually similar to those reported in the first two columns of Fig. 4 have been achieved. However, for high-complexity object (the third column of Fig. 4), the experimental results are not acceptable due to really lower SNR involved in our experimental condition, as discussed above.

### Simulations

All numerical simulation results are obtained by using the commercial software, CST Microwave Studio 2014, and MATLAB 2014 Program Environment. The receiver used in Fig. 5 is an electric-field probe.

### Reconstruction algorithms

The spatial information of the probed object characterized by its reflectivity *O*(*x*, *y*, *z*) is embodied in the one-dimensional frequency-dependent signal, denoted by *s*(*f*), by the spatio-temporal dispersive metasurface, where *f* denotes operational frequency. It can be mathematically reformulated in the compact form as


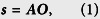


where ***s*** is a column vector representing a collection of frequency-dependent measurements, ***A*** is the measurement operator calibrated using above mentioned point-to-point scanning technique, and ***O*** is a column vector corresponding to the reflectivity of the sampled scene. By applying the traditional minimum least-square reconstruction algorithm, the estimate of ***O*** can be obtained as


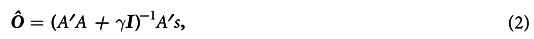


where *γ* is a regularization factor taken as 10^−5^ in our design.

The objects under consideration, as illustrated in Figs 4 and 5 are visually sparse more or less in the spatial domain, such *prior* or feature can be exploited to enhance the reconstruction quality or save measurements by performing a convex optimization problem^24^,^27^,^28^,^43^,^44^. For our case, a sparsity-promoted convex optimization is needed, i.e.,


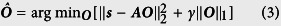


The iteratively reweighting reconstruction algorithm, a popular technique widely used in the community of sparse signal processing, is applied to solve Eq. (3), as demonstrated in Table 1. Here, ***A***′ denotes the conjugate transpose of ***A***, ***O***(*n*) denotes the *n*th element of ***O***, and δ (δ = 10^−5^ is used in this article) is a small positive constant to avoid the singularity when |***O***(*n*)|^2^ vanishes. It is not straightforward to choose suitable values for the artificial parameter γ, although some time-consuming methods, such as L-curve and generalized cross validation approaches, have been proposed. Regarding to the stop criterion, we specify the maximum iteration step heuristically to be 10.

## Additional Information

**How to cite this article**: Wang, L. *et al.* Single-shot and single-sensor high/super-resolution microwave imaging based on metasurface. *Sci. Rep.* **6**, 26959; doi: 10.1038/srep26959 (2016).

## Supplementary Material

Supplementary Information

Supplementary Video 1

## Figures and Tables

**Table 1:** Algorithm procedure solving Eq. (3).

**Input: s, A and** ***γ***
**Output:** ***ô***
**Initializing** ***ô*** **by** ***ô*** = (*A***′*****A*** **+** ***γI***)^−1^***A*****′s.**
**DO until achieving some stop criteria**
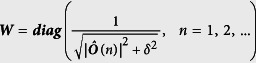
***ô*** = (***A*****′*****A***** + *****γW***)^−1^***A*****′s**
**END DO**
