# Supplementary material for: Single-shot and single-sensor high/super-resolution microwave imaging based on metasurface
Source: Sci Rep. 2016 Jun 1;6:26959. doi: 10.1038/srep26959 (PMC4887868; doi:10.1038/srep26959)
Supplement: Supplementary Information [file srep26959-s1.pdf]

Supplementary Material

Single-shot and single-sensor high/super-resolution microwave imaging based on metasurface

Libo Wang[1], Lianlin Li[1]*, Yunbo Li[2], Hao Chi Zhang[2], &Tie Jun Cui[2]*

[1]School of Electronics Engineering and Computer Science, Peking University, Beijing 100871, China

[2]State Key Laboratory of Millimeter Waves, Southeast University, Nanjing 210096, China

Corresponding authors:

Lianlin Li (lianlin.li@pku.edu.cn); Tie Jun Cui (tjcui@seu.edu.cn)

Video Legend

This video is used to examine the effect on the imaging quality from the distance between the metasurface and the object under consideration. From this video, we observe that the imaging quality will become worse as the distance between the object and the metasurface gets larger, since for the relatively bigger distance the evanescent wave carrying the information of the finer structures of the probed objet will vanish before arriving at the metasurface, and thus cannot be captured by the single-sensor in the far field. In addition, this video is simulated using CST Microwave Studio 2014 in combination with the iteratively reweighting reconstruction algorithm, where the imaged object is the same as that in Fig. 5(a).
